# Characterizing SPASM/twitch Domain-Containing Radical SAM Enzymes by EPR Spectroscopy

**DOI:** 10.1007/s00723-021-01406-2

**Published:** 2021-08-12

**Authors:** Aidin R. Balo, Lizhi Tao, R. David Britt

**Affiliations:** Department of Chemistry, University of California, Davis, CA 95616 USA

## Abstract

Owing to their importance, diversity and abundance of generated paramagnetic species, radical *S*-adenosylmethionine (rSAM) enzymes have become popular targets for electron paramagnetic resonance (EPR) spectroscopic studies. In contrast to prototypic single-domain and thus single-[4Fe–4S]-containing rSAM enzymes, there is a large subfamily of rSAM enzymes with multiple domains and one or two additional iron–sulfur cluster(s) called the SPASM/twitch domain-containing rSAM enzymes. EPR spectroscopy is a powerful tool that allows for the observation of the iron–sulfur clusters as well as potentially trappable paramagnetic reaction intermediates. Here, we review continuous-wave and pulse EPR spectroscopic studies of SPASM/twitch domain-containing rSAM enzymes. Among these enzymes, we will review in greater depth four well-studied enzymes, BtrN, MoaA, PqqE, and SuiB. Towards establishing a functional consensus of the additional architecture in these enzymes, we describe the commonalities between these enzymes as observed by EPR spectroscopy.

## Introduction

**Fig. 1:**
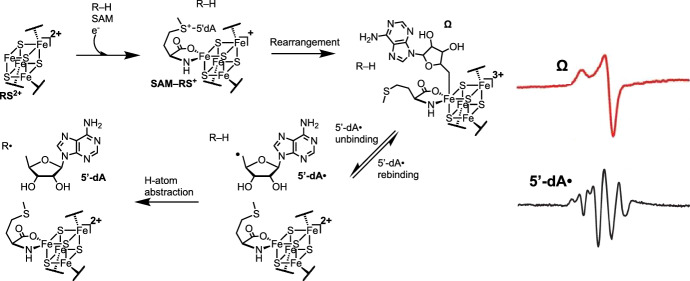
Current radical SAM enzyme initiation mechanism. The RS [4Fe–4S] cluster catalyzes the reductive cleavage of SAM to form L-Met and the reactive 5′-dA• intermediate [6] via the organometallic intermediate Ω [8]. Liberated 5′-dA• abstracts H• from a generic substrate R–H to activate the substrate

With over 570,000 known members spanning all domains of life, the radical *S*-adenosylmethionine (rSAM) enzyme superfamily is the largest known, with most of its members currently uncharacterized [1–4]. The common feature of rSAM enzymes is a canonical [4Fe–4S] cluster, called the rSAM (RS) cluster, usually bound by a CX_3_CX_2_C motif, that catalyzes reductive cleavage of SAM to form L-methionine and a strongly oxidizing 5′-deoxyadenosyl radical (5′-dA•) via the organometallic intermediate Ω (Fig. 1) [5–9]. Both of these intermediate species have been observed by EPR spectroscopy. 5′-dA• usually abstracts a hydrogen atom (H-atom) from substrate to initiate an extremely diverse set of chemical reactions, including the biosynthesis of DNA, cofactor, vitamin, and antibiotics [10–13].

Of the 570,000 rSAM enzyme superfamily members, ~ 165,000 genes (Enzyme Function Initiative—Enzyme Similarity Tool; EFI-EST) encode proteins that possess C-terminal extensions called SPASM domains or twitch domains, which bind auxiliary iron–sulfur cluster(s) [4, 14–19]. The SPASM domain typically binds two auxiliary iron–sulfur clusters (AuxI and AuxII) and is named after the rSAM enzymes involved in the synthesis of Subtilosin, Pyrroloquinoline quinone, Anaerobic Sulfatase, and Mycofactocin. The twitch domain is a truncated SPASM domain and only binds one auxiliary cluster (Aux) [15]. Direct evidence for the catalytic function(s) of any SPASM/twitch domain auxiliary clusters has long remained elusive. While they have been proposed to serve as electron acceptors [20, 21], only recently was the AuxI [4Fe–4S] cluster in the SPASM domain-containing rSAM enzyme SuiB directly observed to accept an electron from the radical intermediate using electron paramagnetic resonance (EPR) spectroscopy [22].

EPR spectroscopy is a powerful technique for characterizing rSAM enzymes among other iron–sulfur cluster-containing and radical-generating enzymes. The hyperfine interaction (HFI) between the paramagnetic centers in rSAM enzymes (e.g., [4Fe–4S]^+^ clusters or trapped paramagnetic intermediates) and nearby magnetic nuclei can be probed using continuous-wave and pulse EPR spectroscopy. Large HFIs in organic radicals can often be observed directly in a continuous-wave EPR spectrum, whereas observing small HFIs in organic radicals and most HFIs in metal-centered paramagnetic species would require pulse EPR techniques such as HYSCORE, ESEEM and ENDOR. Reviews have been written about the studies of rSAM enzymes by continuous-wave and pulse EPR spectroscopy, discussing the spectroscopic features of trapped paramagnetic intermediates and iron–sulfur clusters in various rSAM enzymes [5, 23, 24]. Here, we focus specifically on EPR spectroscopy-based characterizations of SPASM/twitch domain-containing rSAM enzymes, and review in greater depth the studies of two twitch domain-containing rSAM enzymes, BtrN and MoaA, as well as those of two SPASM domain-containing rSAM enzymes, PqqE and SuiB.

## Twitch Domain-Containing Radical SAM Enzymes

**Fig. 2:**
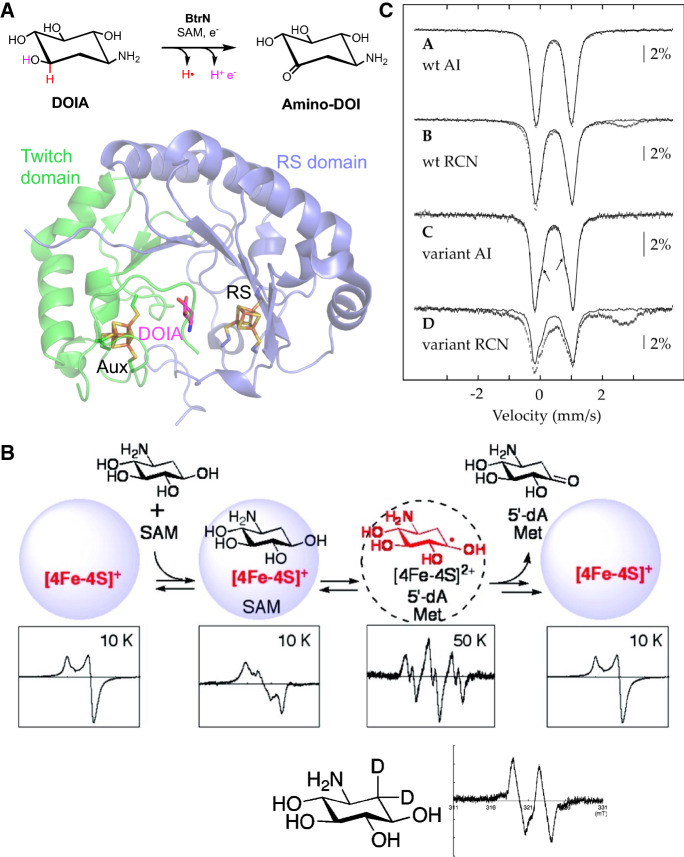
BtrN. **a** Reaction and structure of twitch domain-containing rSAM enzyme BtrN (PDB ID: 4M7T). **b** EPR study of BtrN [30]. The characteristically axial RS cluster became rhombic upon binding SAM and substrate DOIA. A radical intermediate of DOIA following H-atom abstraction was trapped and observed by EPR. Its identity was confirmed using the isotopologue [2,2-^2^H_2_]DOIA, which resulted in loss of observable hyperfine splitting. **c** Mössbauer spectra of the wild type as isolated (wt AI), reconstituted (wt RCN), RS cluster knockout C16A/C20A/C23A as isolated (variant AI), and reconstituted (wt RCN) [20]. The Mössbauer spectra of the knockout mutant revealed an additional cluster, which has a low reduction potential, such that it is therefore EPR silent in dithionite-reduced samples. It was not known to exist during the study of EPR study in part B. It should be noted that the crystal structures of BtrN were solved after the EPR and Mössbauer studies

Butirosin is an antibiotic with broad Gram-negative and Gram-positive inhibitory and some bactericidal properties [25]. BtrN is a twitch domain-containing rSAM enzyme which catalyzes the two-electron oxidation of 2–deoxy–*scyllo*–inosamine (DOIA) to 3–amino–2,3–dideoxy–*scyllo*–inosose (amino-DOI; Fig. 2A) in the biosynthetic pathway of butirosin [26–29]. Early mechanistic studies trapped a radical intermediate of DOIA with *g* = 2.0025 formed after H-atom abstraction by 5′-dA•, which was confirmed by selective isotopic labeling of the substrate DOIA (Fig. 2B) [30]. In the same study, the EPR spectra of the characteristically axial RS cluster and the more rhombic SAM-bound RS cluster are also presented.

At that time, it was not known that BtrN contained a second [4Fe–4S] cluster, i.e., the Aux cluster. This Aux cluster was first observed by Mössbauer spectroscopy using a mutant with RS cluster knocked out (C16A/C20A/C23A), which possesses an additional [4Fe–4S] cluster (Fig. 2C) [20]. A subsequent X-ray crystal structural study of BtrN confirmed that this Aux [4Fe–4S] cluster is ligated by four cysteine residues [27]. However, this Aux [4Fe–4S] cluster is not observable by EPR using dithionite as the reducing agent (with the reduction potential − 660 mV vs. NHE at pH = 7), suggesting that this Aux [4Fe–4S] exhibits a reduction potential lower than − 660 mV. The low potential of this Aux [4Fe–4S] cluster was later confirmed to be − 765 mV vs. NHE in a protein electrochemistry study [31]. The low potential of this Aux cluster leads the authors to propose that the Aux cluster may serve as the final electron acceptor of the radical intermediate during the oxidation reaction [20]. However, direct evidence for electron transfer from the radical intermediate to the Aux cluster, e.g., the EPR spectrum of the AuxI cluster which is reduced by the radical intermediate, has not been reported yet.

**Fig. 3:**
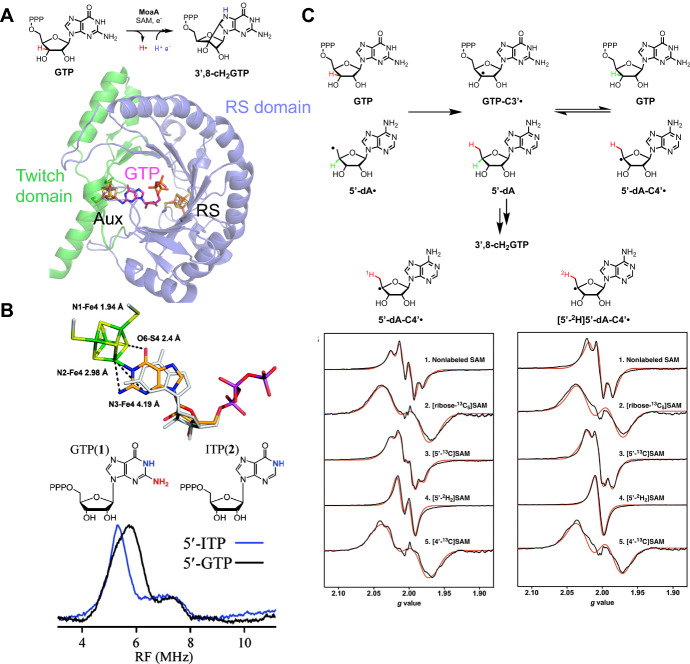
MoaA. **a** Reaction and structure of twitch domain-containing rSAM enzyme MoaA (PDB ID: 2FB3). **b** ENDOR study of the interaction between the MoaA Aux cluster and substrate GTP using the RS cluster knockout mutant C24S/C28S/C31S [35]. The study used the inosine triphosphate (ITP), an analogue of GTP lacking the N2 amino group, to position the substrate relative to the cluster. The ENDOR study found that the guanine N1 binds the Aux cluster with a distance of 1.94 Å, whereas the crystal structure (gray; PDB ID: 2FB3) modeled the N2 amino group as the closest group to the Aux cluster open iron site with a distance of 2.4 Å. **c** A mechanistic EPR study of MoaA traps a radical intermediate [36]. The hyperfine splitting in the spectra of the intermediate varied when using either natural abundance GTP or [3′-^2^H]GTP with nonlabeled SAM. In addition, using various SAM isotopologues also affected the line shape. The authors concluded that the intermediate was 5′-dA-C4′•, forming through an H-atom abstraction of the quenched 5′-dA by GTP-C3′•

MoaA is another twitch domain-containing rSAM enzyme which catalyzes the cyclization of GTP to 3′,8-cH_2_GTP (Fig. 3A) in the biosynthetic pathway of molybdopterin, an important molybdenum- or tungsten-based cofactor necessary for a wide variety of enzymatic reactions [32, 33]. An X-ray crystal structural study shows that MoaA harbors a canonical RS [4Fe–4S] cluster in the N-terminal domain and an Aux [4Fe–4S] cluster in the C-terminal domain [34]. This Aux [4Fe–4S] cluster is ligated by three cysteine residues, leaving an open Fe site. The observable EPR spectrum of the MoaA mutant with its RS cluster knocked out and using dithionite as the reducing agent [35] suggests that this Aux [4Fe–4S] cluster has a reduction potential more positive than − 660 mV vs. NHE at pH = 7, which is in contrast to the low-potential Aux cluster in BtrN (*vide supra*).

A GTP-bound MoaA crystal structure showed that the substrate GTP binds to the open iron site on the Aux [4Fe–4S] cluster through either or both the guanosine N1 and the amine N2 [34]. However, the electron density of the ribose and purine of GTP is not well-defined, showing that the N1 and N2 are likely around 2.8 Å and 2.4 Å, respectively, away from the open iron site of the Aux cluster, both of which are too far for bonding [34]. A subsequent study used a pulse EPR technique called electron–nuclear double resonance (ENDOR) spectroscopy to investigate the binding mode of GTP to the open iron site of the Aux cluster (Fig. 3B) more precisely via analysis of the HFIs between ^14/15^ N and the paramagnetic Aux cluster [35]. The authors reported ENDOR spectra of MoaA RS cluster knockout mutant C24S/C28S/C31S bound to either GTP or inosine triphosphate (ITP), a functional substrate analogue of GTP missing the N2-containing amine group. Based on the HFIs measured by ENDOR spectroscopy, the distances from the unique AuxI iron site to the guanosine N1 and amine N2 were calculated to be 1.94 Å and 2.98 Å, respectively. The similar *A*_iso_(^14^ N) values (3.6 MHz for GTP, 3.5 MHz for ITP) suggested that the common guanosine N1 binds to the open iron site (rather than the amine N2 of GTP) via tautomerization to form an enol. Thus, the Aux cluster may play an important role other than oxidizing the radical intermediate.

During MoaA catalytic reaction, an unusual radical intermediate, 5′-dA-C4′•, was later discovered and reported, which is in contrast to the standard 5′-dA-C5′• (or simply 5′-dA•) and is formed in a shunt pathway through an H-atom abstraction of the quenched 5′-dA at C4′ by the substrate radical intermediate GTP-C3′• (Fig. 3C) [36]. The EPR hyperfine splitting of the 5′-dA-C4′• intermediate varied when using either natural abundance GTP or [3′-^2^H]GTP with non-labeled SAM, suggesting that H3′ of GTP is abstracted by 5′-dA• to initiate the reaction. In addition, using various SAM isotopologues also affected the EPR splitting pattern and thus the line shape, further confirming that the intermediate is indeed a SAM-based radical, rather than a GTP-based radical [36].

While structurally similar, we note three fundamental and important differences between MoaA and its relative BtrN. First, the Aux cluster of MoaA has one open Fe site [34], to which the substrate GTP binds, whereas, the Aux cluster of BtrN is ligated by four cysteine residues [27]. Second, EPR spectroscopy suggests that MoaA has a high-potential Aux cluster which is readily reducible by dithionite [35], whereas BtrN has a low-potential Aux cluster relative to dithionite [20, 30, 31]. Third, the reaction catalyzed by BtrN results in a two-electron oxidation from substrate to product, whereas, the reaction catalyzed by MoaA results in a net-zero loss of electrons [33].

### SPASM Domain-Containing Radical SAM Enzymes

**Fig. 4:**
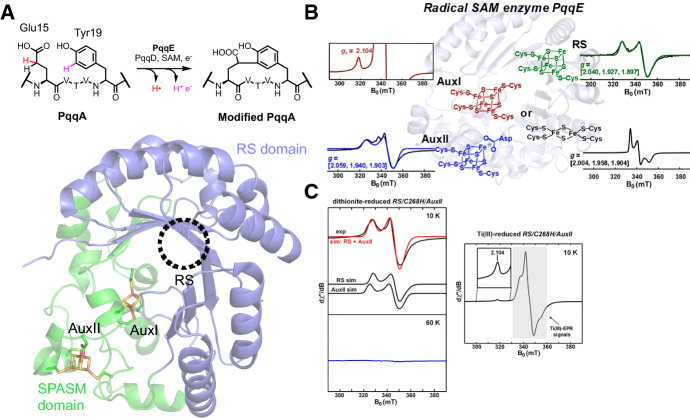
PqqE. **a** Reaction and structure of SPASM domain-containing rSAM enzyme PqqE (PDB ID: 6C8V). **b** A detailed EPR characterizations of the iron–sulfur clusters in PqqE [42]. While the RS and AuxII clusters were found to be high-potential [4Fe–4S] clusters, the AuxI cluster was found to be a mixture of a low-potential [4Fe–4S] and a high-potential [2Fe–2S]. **c** A subsequent publication found that a mutation of one of the AuxI-binding cysteine residues to histidine (C268H) resulted in no [2Fe–2S] cluster formation, which is observable by EPR at 60 K [43]. However, the low-potential [4Fe–4S] AuxI signal was observable when using the strong reducing agent Ti(III) citrate

PqqE is a SPASM domain-containing rSAM enzyme which catalyzes the C–C bond formation between Glu15 and Tyr19 of substrate peptide PqqA (numbering varies by organism) via a two-electron oxidation (Fig. 4A) in the biosynthetic pathway of pyrroloquinoline quinone, a redox-active cofactor produced by microorganisms and acquired by humans through diet [37–39]. Early studies of PqqE reported EPR spectra of iron–sulfur clusters [40] and the clusters when PqqE is bound to its peptide chaperone protein PqqD [41]. Of its three clusters, the AuxI cluster was initially observed by crystallography as a [2Fe–2S] cluster [16], but was later confirmed by EPR to exist as a heterogeneous mixture of a high-potential [2Fe–2S] cluster and a low-potential [4Fe–4S] cluster (Fig. 4B) [42]. The low-potential [4Fe–4S] AuxI cluster can only be reduced by the strong reducing agents Ti(III) citrate or Eu(II)-DTPA with the reduction potential < − 800 mV vs NHE. Only the *g*_1_ of AuxI (2.104) could be observed, because these strong reducing agents are themselves paramagnetic and in excess obscured the region overlapping with AuxI. Possible biological reasons for observing both [4Fe–4S] and [2Fe–2S] AuxI clusters were discussed in a subsequent publication, such as a higher abundance of [2Fe–2S] in aerobes and facultative anaerobes [43]. Moreover, the authors show that an interesting mutant with a substitution at one of the cysteine residues binding the AuxI cluster for a histidine (C268H) does not exhibits the [2Fe–2S]^+^ signal observed at 60 K, while the [4Fe–4S]^+^ AuxI signal is present (Fig. 4C). While this mutant is incapable of catalytic function of C–C bond formation, it has yet to be confirmed which iron–sulfur cluster species is the final electron acceptor.

**Fig. 5:**
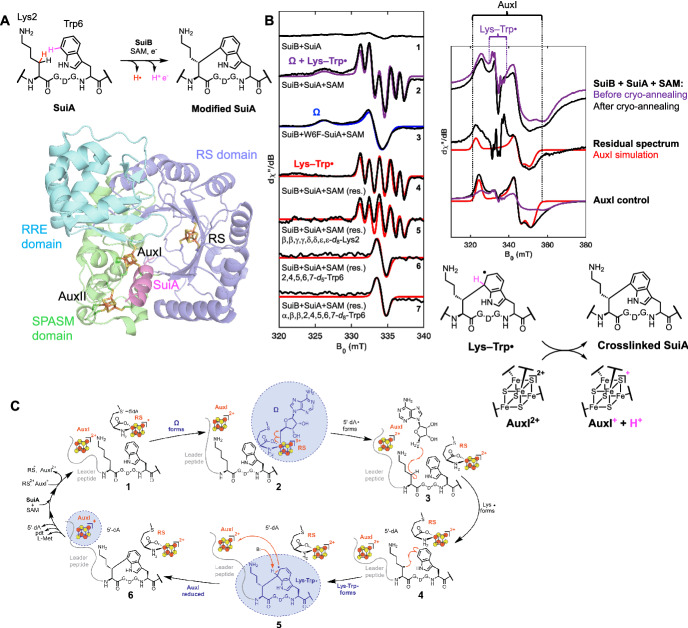
SuiB. **a** Reaction and structure of SPASM domain-containing rSAM enzyme SuiB (PDB ID: 5V1T). **b** An EPR study of trapped paramagnetic intermediates in SuiB, most notably the Lys–Trp• intermediate, which was confirmed using SuiA isotopologues (22). The organometallic intermediate Ω was also observed. In the same study, the unpaired electron from the Lys–Trp• intermediate can be transferred to the AuxI cluster by cryo-annealing, revealing AuxI as the electron acceptor. **c** A detailed mechanism of the catalytic cycle for SuiB with EPR-observed intermediates shown in blue

SuiB is another SPASM domain-containing rSAM enzyme which catalyzes the C–C bond formation between Lys2 and Trp6 of substrate peptide SuiA via a two-electron oxidation (Fig. 5A) in the biosynthetic pathway of the Streptide, a cyclic peptide and novel streptococcal natural product [44, 45]. The three [4Fe–4S] clusters in SuiB were observed by crystallography [14] and by EPR spectroscopy via cluster knockout mutants [45]. Although not all of the individual *g*-tensors were well resolved by X-band (9.4 GHz) spectra, Q-band (34.0 GHz) EPR spectroscopy was able to identify the *g*-tensors of three [4Fe–4S] clusters [22]. During SuiB enzymatic turnover, in addition to a semi-stable organometallic intermediate Ω, a one-electron oxidized radical intermediate Lys–Trp• has been trapped and characterized by EPR spectroscopy (Fig. 5B) [22]. The identity of Lys–Trp• was confirmed using substrate SuiA isotopologues. The intermediate confirmed that SuiB catalyzes a radical electrophilic aromatic substitution (rEAS) reaction. The reaction catalyzed by PqqE is likely catalyzed through a rEAS reaction analogous to the reaction catalyzed by SuiB. In SuiB, it was found that anaerobic cryo-annealing at 200 K results in the disappearance of the Lys–Trp• signal and the appearance of the AuxI signal, suggesting that the electron is transferred directly from the radical intermediate to the AuxI cluster and providing the first direct evidence for the electron acceptor role of the AuxI cluster [22]. Moreover, the AuxI cluster was found to be a low-potential cluster only when the substrate SuiA was bound to SuiB. At the same time, upon binding SuiA, the reduction potential of the RS cluster became more positive, suggesting that the electron captured by AuxI may be either directly or indirectly transferred back to the RS cluster for subsequent reactions. These findings and paramagnetic intermediates were summarized as a detailed catalytic mechanism for SuiB (Fig. 5C). EPR spectra of [4Fe–4S] clusters of two orthologues of SuiB, namely StrB [44] and AgaB [45], as well as another Lys–Trp crosslinking enzyme, WgkB [46], have also been reported.

MftC, another SPASM domain-containing rSAM enzyme, performs an oxidative decarboxylation on N-terminal Tyr30 of MftA followed by a C–C crosslinking reaction between the C_α_–C_β_ unsaturated Tyr30 and the penultimate Val29 residue in a subsequent SAM-dependent turnover [47, 48]. The [4Fe–4S] clusters of MftC have been measured by EPR spectroscopy while varying the presence of its binding partners: SAM, MftA (substrate peptide), and MftB (peptide chaperone). [49].

EPR spectra of [4Fe–4S] clusters have also been reported on other SPASM domain-containing rSAM enzymes that catalyze the S–C_α_ and S–C_β_ thioether peptide crosslinking reactions, including Tte1186 (S–C_α_) [50], RumMc1 (S–C_α_) [51], AlbA (S–C_α_) [13], GggB (S–C_α_) [52], and NxxcB (S–C_β_) [53]. Many SPASM domain rSAM enzymes, including PqqE, SuiB (and its orthologues), WgkB, MftC, Tte1186, RumMc1, AlbA, GggB, and NxxcB, are part of biosynthetic pathways to produce ribosomally synthesized and post-translationally modified peptides (RiPPs) [15, 54–58].

## Outlook for Studying SPASM/twitch Domain-Containing Radical SAM Enzymes by EPR Spectroscopy

rSAM enzymes constitute a massive and continuously growing superfamily of enzymes capable of novel chemistries that are often not well understood upon discovery. While the effort to characterize the general mechanisms of rSAM enzymes is underway, the nuances of the great number of SPASM/twitch domain-containing rSAM enzymes relative to single-domain (and single-[4Fe–4S]) rSAM enzymes must also be understood. EPR spectroscopy, among other techniques, has played a crucial role in mechanistic characterizations in rSAM enzymes. Here, we focused on discussing four examples SPASM/twitch domain-containing rSAM enzymes characterized by EPR spectroscopy.

BtrN, PqqE and SuiB all perform two-electron oxidation reactions. The AuxI cluster in SuiB was observed to accept an electron from the radical intermediate Lys–Trp• [22]. By extension, the Aux cluster of BtrN and the AuxI of PqqE are likely also electron acceptors from their respective radical intermediates. All three of these [4Fe–4S] Aux/AuxI clusters have been observed by EPR to be low-potential clusters. The case of MoaA is exceptional, in that the degree of unsaturation from the substrate is preserved in the product (i.e., no net loss of electrons), and its Aux cluster has a more positive potential compared to BtrN, PqqE and SuiB. It is possible that in this case the Aux cluster serves another function, such as: (i) performing a catalytic role via the direct interaction with the substrate GTP, (ii) providing the electron needed to quench the radical intermediate, or (iii) simply being conserved as a vestigial cluster.

Understanding the components of these enzymes is important for realizing their applications, such as new avenues for drug (bio)synthesis and protein engineering efforts. EPR spectroscopy is unparalleled in its ability to directly observe, identify and characterize fleeting paramagnetic intermediates, as well as the complicated iron–sulfur clusters in rSAM enzymes. Despite a great abundance of SPASM/twitch domain-containing rSAM enzymes, very few have been investigated by EPR spectroscopy. EPR studies of additional systems are needed to cement the role(s) of the SPASM/twitch domains in catalysis. Moreover, for SPASM domain-containing rSAM enzymes, the role of AuxII cluster remains unclear. Perhaps studying the enzymatic reactions under conditions closer to the in vivo ones, such as using biological reductants [59], may reveal important mechanistic details.

## References

[1] Sofia HJ, Chen G, Hetzler BG, Reyes-Spindola JF, Miller NE (2001). Radical SAM, a novel protein superfamily linking unresolved steps in familiar biosynthetic pathways with radical mechanisms: functional characterization using new analysis and information visualization methods. Nucleic. Acids. Res..

[2] Caruso A, Martinie RJ, Bushin LB, Seyedsayamdost MR (2019). Macrocyclization via an Arginine-Tyrosine crosslink broadens the reaction scope of radical. J. Am. Chem. Soc..

[3] Landgraf BJ, McCarthy EL, Booker SJ (2016). Radical *S*-Adenosylmethionine enzymes in human health and disease. Annu. Rev. Biochem..

[4] M.R. Seyedsayamdost, A. Caruso, K.M. Davis, The chemistry and structural enzymology of RiPP-modifying radical SAM metalloenzymes. Invited Book Chapter Submission: “Comprehensive natural products III*”* Edited by Tadhg Begley and Ben Liu. Elsevier, 49–64 (2020)

[5] Broderick JB, Duffus BR, Duschene KS, Shepard EM (2014). Radical *S*-adenosylmethionine enzymes. Chem. Rev..

[6] Sayler RI (2019). Trapping and electron paramagnetic resonance characterization of the 5'dAdo. ACS. Cent. Sci..

[7] Yang H (2019). The elusive 5'-deoxyadenosyl radical: captured and characterized by electron paramagnetic resonance and electron nuclear double resonance spectroscopies. J. Am. Chem. Soc..

[8] Horitani M (2016). Radical SAM catalysis via an organometallic intermediate with an Fe-[5'-C]-deoxyadenosyl bond. Science.

[9] Byer AS (2018). Paradigm shift for radical *S*-adenosyl-l-methionine reactions: the organometallic intermediate Ω is central to catalysis. J. Am. Chem. Soc..

[10] Berkovitch F, Nicolet Y, Wan JT, Jarrett JT, Drennan CL (2004). Crystal structure of biotin synthase, an *S*-adenosylmethionine-dependent radical enzyme. Science.

[11] Grove TL (2011). A radically different mechanism for *S*-adenosylmethionine-dependent methyltransferases. Science.

[12] Klinman JP, Bonnot F (2014). Intrigues and intricacies of the biosynthetic pathways for the enzymatic quinocofactors: PQQ, TTQ, CTQ, TPQ, and LTQ. Chem. Rev..

[13] Flühe L (2012). The radical SAM enzyme AlbA catalyzes thioether bond formation in subtilosin A. Nat. Chem. Biol..

[14] Davis KM (2017). Structures of the peptide-modifying radical SAM enzyme SuiB elucidate the basis of substrate recognition. Proc. Natl. Acad. Sci. U S A.

[15] Grell TA, Goldman PJ, Drennan CL (2015). SPASM and twitch domains in *S*-adenosylmethionine (SAM) radical enzymes. J. Biol. Chem..

[16] Barr I (2018). X-ray and EPR characterization of the auxiliary Fe-S clusters in the radical SAM enzyme PqqE. Biochemistry.

[17] Grell TAJ (2018). Structural and spectroscopic analyses of the sporulation killing factor biosynthetic enzyme SkfB, a bacterial AdoMet radical sactisynthase. J. Biol. Chem..

[18] Grove TL (2017). Structural insights into thioether bond formation in the biosynthesis of Sactipeptides. J. Am. Chem. Soc..

[19] Haft DH, Basu MK (2011). Biological systems discovery in silico: radical *S*-adenosylmethionine protein families and their target peptides for posttranslational modification. J. Bacteriol..

[20] Grove TL, Ahlum JH, Sharma P, Krebs C, Booker SJ (2010). A consensus mechanism for radical SAM-dependent dehydrogenation? BtrN contains two [4Fe-4S] clusters. Biochemistry.

[21] Lanz ND, Booker SJ (2015). Auxiliary iron-sulfur cofactors in radical SAM enzymes. Biochim. Biophys. Acta..

[22] Balo AR (2021). Trapping a cross-linked lysine-tryptophan radical in the catalytic cycle of the radical SAM enzyme SuiB. Proc Natl Acad Sci U S A.

[23] Stich TA, Myers WK, Britt RD (2014). Paramagnetic intermediates generated by radical *S*-adenosylmethionine (SAM) enzymes. Acc. Chem. Res..

[24] Broderick WE, Hoffman BM, Broderick JB (2018). Mechanism of radical initiation in the radical *S*-adenosyl-l-methionine superfamily. Acc. Chem. Res..

[25] Heifetz CL, Fisher MW, Chodubski JA, DeCarlo MO (1972). Butirosin, a new aminoglycosidic antibiotic complex: antibacterial activity in vitro and in mice. Antimicrob. Agents. Chemother..

[26] Yokoyama K, Numakura M, Kudo F, Ohmori D, Eguchi T (2007). Characterization and mechanistic study of a radical SAM dehydrogenase in the biosynthesis of butirosin. J. Am. Chem. Soc..

[27] Goldman PJ, Grove TL, Booker SJ, Drennan CL (2013). X-ray analysis of butirosin biosynthetic enzyme BtrN redefines structural motifs for AdoMet radical chemistry. Proc. Natl. Acad. Sci. U S A.

[28] Li Y, Llewellyn NM, Giri R, Huang F, Spencer JB (2005). Biosynthesis of the unique amino acid side chain of butirosin: possible protective-group chemistry in an acyl carrier protein-mediated pathway. Chem. Biol..

[29] Tamegai H (2002). Identification of l-glutamine: 2-deoxy-scyllo-inosose aminotransferase required for the biosynthesis of butirosin in Bacillus circulans. J. Antibiot. (Tokyo).

[30] Yokoyama K, Ohmori D, Kudo F, Eguchi T (2008). Mechanistic study on the reaction of a radical SAM dehydrogenase BtrN by electron paramagnetic resonance spectroscopy. Biochemistry.

[31] Maiocco SJ, Grove TL, Booker SJ, Elliott SJ (2015). Electrochemical resolution of the [4Fe-4S] centers of the AdoMet radical enzyme BtrN: evidence of proton coupling and an unusual, low-potential auxiliary cluster. J. Am. Chem. Soc..

[32] Leimkühler S, Wuebbens MM, Rajagopalan KV (2011). The history of the discovery of the molybdenum cofactor and novel aspects of its biosynthesis in bacteria. Coord. Chem. Rev..

[33] Hover BM, Loksztejn A, Ribeiro AA, Yokoyama K (2013). Identification of a cyclic nucleotide as a cryptic intermediate in molybdenum cofactor biosynthesis. J. Am. Chem. Soc..

[34] Hänzelmann P, Schindelin H (2004). Crystal structure of the *S*-adenosylmethionine-dependent enzyme MoaA and its implications for molybdenum cofactor deficiency in humans. Proc. Natl. Acad. Sci. U S A.

[35] Lees NS (2009). ENDOR spectroscopy shows that guanine N1 binds to [4Fe-4S] cluster II of the *S*-adenosylmethionine-dependent enzyme MoaA: mechanistic implications. J. Am. Chem. Soc..

[36] Pang H (2020). Mechanism of rate acceleration of radical C-C bond formation reaction by a radical SAM GTP 3',8-cyclase. J. Am. Chem. Soc..

[37] Puehringer S, Metlitzky M, Schwarzenbacher R (2008). The pyrroloquinoline quinone biosynthesis pathway revisited: a structural approach. BMC. Biochem..

[38] Magnusson OT, Toyama H, Saeki M, Schwarzenbacher R, Klinman JP (2004). The structure of a biosynthetic intermediate of pyrroloquinoline quinone (PQQ) and elucidation of the final step of PQQ biosynthesis. J. Am. Chem. Soc..

[39] Zhu W, Klinman JP (2020). Biogenesis of the peptide-derived redox cofactor pyrroloquinoline quinone. Curr. Opin. Chem. Biol..

[40] Wecksler SR (2009). Pyrroloquinoline quinone biogenesis: demonstration that PqqE from Klebsiella pneumoniae is a radical *S*-adenosyl-l-methionine enzyme. Biochemistry.

[41] Wecksler SR (2010). Interaction of PqqE and PqqD in the pyrroloquinoline quinone (PQQ) biosynthetic pathway links PqqD to the radical SAM superfamily. Chem. Commun. (Camb).

[42] Tao L, Zhu W, Klinman JP, Britt RD (2019). Electron paramagnetic resonance spectroscopic identification of the Fe-S clusters in the SPASM domain-containing radical SAM enzyme PqqE. Biochemistry.

[43] Zhu W (2020). Structural properties and catalytic implications of the SPASM domain iron-sulfur clusters in. J. Am. Chem. Soc..

[44] Schramma KR, Bushin LB, Seyedsayamdost MR (2015). Structure and biosynthesis of a macrocyclic peptide containing an unprecedented lysine-to-tryptophan crosslink. Nat. Chem..

[45] Schramma KR, Seyedsayamdost MR (2017). Lysine-Tryptophan-crosslinked peptides produced by radical SAM enzymes in pathogenic streptococci. ACS. Chem. Biol..

[46] Bushin LB, Clark KA, Pelczer I, Seyedsayamdost MR (2018). Charting an unexplored streptococcal biosynthetic landscape reveals a unique peptide cyclization motif. J. Am. Chem. Soc..

[47] Khaliullin B, Ayikpoe R, Tuttle M, Latham JA (2017). Mechanistic elucidation of the mycofactocin-biosynthetic radical. J. Biol. Chem..

[48] Bruender NA, Bandarian V (2016). The radical *S*-adenosyl-l-methionine enzyme MftC catalyzes an oxidative decarboxylation of the C-terminus of the MftA peptide. Biochemistry.

[49] Ayikpoe R (2019). Spectroscopic and electrochemical characterization of the Mycofactocin biosynthetic protein, MftC, provides insight into its redox flipping mechanism. Biochemistry.

[50] Bruender NA, Wilcoxen J, Britt RD, Bandarian V (2016). Biochemical and spectroscopic characterization of a radical *S*-adenosyl-l-methionine enzyme involved in the formation of a peptide thioether cross-link. Biochemistry.

[51] Roblin C (2020). The unusual structure of ruminococcin C1 antimicrobial peptide confers clinical properties. Proc. Natl. Acad. Sci. U S A.

[52] Bushin LB, Covington BC, Rued BE, Federle MJ, Seyedsayamdost MR (2020). Discovery and biosynthesis of streptosactin, a sactipeptide with an alternative topology encoded by commensal bacteria in the human microbiome. J. Am. Chem. Soc..

[53] Caruso A, Bushin LB, Clark KA, Martinie RJ, Seyedsayamdost MR (2019). Radical approach to enzymatic β-thioether bond formation. J. Am. Chem. Soc..

[54] Ortega MA, van der Donk WA (2016). New insights into the biosynthetic logic of ribosomally synthesized and post-translationally modified peptide natural products. Cell. Chem. Biol..

[55] Montalbán-López M (2021). New developments in RiPP discovery, enzymology and engineering. Nat. Prod. Rep..

[56] Arnison PG (2013). Ribosomally synthesized and post-translationally modified peptide natural products: overview and recommendations for a universal nomenclature. Nat. Prod. Rep..

[57] Hetrick KJ, van der Donk WA (2017). Ribosomally synthesized and post-translationally modified peptide natural product discovery in the genomic era. Curr. Opin. Chem. Biol..

[58] Benjdia A, Balty C, Berteau O (2017). Radical SAM enzymes in the biosynthesis of ribosomally synthesized and post-translationally modified peptides (RiPPs). Front. Chem..

[59] Bruender NA, Young AP, Bandarian V (2015). Chemical and biological reduction of the radical SAM enzyme 7-Carboxy-7-deazaguanine [corrected] synthase. Biochemistry.

